# Periodontal inflammation in patients with polymyalgia rheumatica

**DOI:** 10.1007/s11845-026-04413-z

**Published:** 2026-04-23

**Authors:** Irada Ibramkhalilova, Muhammed Aslan, Suleyman Ziya Senyurt, Fatih Albayrak, Ipek Kocer, Buse Cengiz, Orhan Zengin, Bunyamin Kisacik

**Affiliations:** 1 Department of Internal Medicine, Division of Rheumatology, Faculty of Medicine, Sanko University, Gaziantep, Turkey; 2 Department of Internal Medicine, Faculty of Medicine, Sanko University, Gaziantep, Turkey; 3 Department of Periodontology, Faculty of Dentistry, Gaziantep University, Gaziantep, Turkey; 4 Department of Internal Medicine, Division of Rheumatology, Faculty of Medicine, Gaziantep University, Gaziantep, Turkey; 5 Department of Medical Microbiology, Sanko University School of Medicine, Gaziantep, Turkey

**Keywords:** Polymyalgia rheumatica, Periodontal disease, Inflammation, Gingivitis, Periodontitis

## Abstract

**Background:**

Polymyalgia rheumatica (PMR) is an inflammatory disorder affecting individuals aged ≥50 years, in which pro-inflammatory cytokines play a key role and may contribute to periodontal inflammation.

**Aim:**

This study aimed to investigate the frequency and severity of periodontal disease in patients with PMR compared with healthy controls.

**Methods:**

The study included 48 patients diagnosed with PMR and 52 age- and gender-matched systemically healthy individuals as controls. Periodontal status was assessed based on probing depth (PD), clinical attachment level (CAL), plaque index (PI), bleeding on probing (BoP), and gingival index (GI). Periodontal diagnoses were categorized as Stage I or II periodontitis, gingivitis, or healthy periodontium.

**Results:**

Among PMR patients, 11 had Stage I periodontitis, 3 had Stage II periodontitis, 30 had gingivitis, and 4 were periodontally healthy. In the control group, 9 had Stage I periodontitis, 3 had Stage II periodontitis, 34 had gingivitis, and 6 were healthy. While no statistically significant differences were observed between the groups in PPD, CAL, or PI values, both PPD and CAL were higher in the PMR group. BoP was significantly higher in the PMR group compared to controls (58.3±34.7% vs 37.8±25.3%, *p* = 0.001). Similarly, GI scores were significantly higher in PMR patients (1.56±0.50 vs 1.27±0.54, *p* = 0 .007).

**Conclusions:**

Considering the similarities between PMR and rheumatoid arthritis in terms of systemic inflammatory pathways, our results support the hypothesis that PMR, like rheumatoid arthritis, may also be linked to an increased risk of periodontal disease.

## Introduction

Polymyalgia rheumatica (PMR) is an inflammatory disease characterized by proximal muscle pain and stiffness, typically seen in individuals aged 50 and older. In some patients, this clinical picture may be accompanied by peripheral arthritis. Although the exact cause of the disease is not known, it is understood that pro-inflammatory cytokines play an important role in its pathogenesis [1]. Significant increases in the levels of mediators observed in rheumatoid arthritis, such as interleukin-6 (IL-6), interleukin-1β (IL-1β), and tumor necrosis factor-alpha (TNF-α), have been detected [2]. This inflammatory response contributes to the onset of clinical symptoms of PMR.

Periodontal diseases are chronic inflammatory diseases that affect the supporting tissues of the teeth and are generally associated with plaque accumulation and subgingival bacterial microflora [3, 4]. In the early stages, it is in the form of gingivitis, where tissue damage is limited to the soft tissues, but in the later stages, it can progress to periodontitis, leading to serious consequences such as connective tissue loss and alveolar bone destruction. It is well known that genetic factors, smoking, inadequate oral hygiene, and systemic inflammatory diseases play a role in the development of periodontal diseases [5]. It has been reported that the prevalence of periodontal disease increases in inflammatory diseases such as rheumatoid arthritis (RA), psoriatic arthritis (PsA), Behçet’s disease, and familial Mediterranean fever (FMF) [6, 7]. It is thought that systemic inflammation can trigger periodontal diseases. However, there is no study in the literature evaluating the relationship between PMR and periodontal diseases. The main objective of this study is to investigate the frequency of periodontal disease in PMR patients.

## Material and method

This research was designed as a cross-sectional study to evaluate the prevalence of periodontal disease in patients with polymyalgia rheumatica (PMR). This study reviewed the records of 48 patients who had been diagnosed with new-onset polymyalgia rheumatica (PMR) according to the 2012 European League Against Rheumatism (EULAR)/American College of Rheumatology (ACR) provisional classification criteria [8, 9]., along with 52 age- and sex-matched systemically healthy control individuals. All participants had been referred to two different tertiary rheumatology clinics.

Standardized periapical and/or panoramic radiographs were obtained for all participants, and bone loss was evaluated according to the 2017 World Workshop criteria.

### Inclusion and exclusion criteria

#### Inclusion criteria

- For the PMR group: Aged 50 and above, diagnosed with PMR according to the 2012 EULAR/ACR criteria, and not having started treatment,
- For the control group: Being systemically healthy, having applied to the periodontology clinic for periodontal evaluation.

#### Exclusion criteria

- Having previously received steroid or other immunosuppressive treatment,
- Diagnosis of diabetes mellitus,
- Smoking,
- Having undergone periodontal treatment in the last 3 months,
- Systemic diseases requiring antibiotic use,
- Other systemic diseases that can affect periodontal condition (cardiovascular diseases, osteoporosis, etc.)

### Clinical evaluation

The diagnosis of PMR and the assessment of disease activity were conducted by experienced rheumatologists. Disease activity was assessed using the validated PMR Activity Score (PMR-AS) [10]. The activity score of all PMR patients was measured at 17 and bove.

### Periodontal evaluation

All participants’ periodontal examinations were conducted by a single periodontist (S.Z.S.) who was informed about the study protocol but was blinded to the distribution of patients into groups. For standardization, the following clinical parameters were recorded using the Williams periodontal probe (Hu-Friedy, Chicago, Illinois, USA):

- Gingival Index (GI): evaluated according to the Löe index. S coring: 0 = healthy gum; 1 = mild inflammation, color change, edema, no bleeding; 2 = moderate inflammation, redness, edema, and bleeding on probing; 3 = severe inflammation, pronounced redness, edema, ulceration, and tendency for spontaneous bleeding [11].
- Plaque Index (PI): According to the Silness & Löe index, it has been evaluated on the four surfaces of the teeth (buccal, lingual, mesial, and distal). Scoring: 0 = no plaque; 1 = plaque not visible on the tooth surface but detectable when scraped with a probe; 2 = thin plaque visible on the tooth surface; 3 = dense plaque accumulation on the tooth surface [11].
- Probing Pocket Depth (PPD): the value measured between the free gingival margin and the periodontal pocket base using a periodontal probe as a diagnostic tool, indicating the healthy or compromised tissues of the gum and changes in the position of the gum [12]. The measurements taken were rounded to the nearest millimeter.
- Clinical Attachment Level (CAL): The distance between the cemento-enamel junction and the periodontal pocket base was measured from the same six points. It is one of the most important parameters used in the diagnosis of periodontal disease and the evaluation of treatment outcomes [13].
- Bleeding on probing (BoP): The probing procedure is performed by gently moving a periodontal probe within the periodontal pocket or gingival sulcus, and bleeding is evaluated [13]. The base of the pocket was scored as positive (+) or negative (-) based on the presence or absence of bleeding in the sulcus 10 s after probing, in order to determine the inflammatory condition of the pocket epithelium. The BoP index was calculated for each patient using the following equation: Number of bleeding surfaces / total number of tooth surfaces X 100.

### Periodontal disease classification

Periodontal diagnoses were made according to the updated classification of periodontal and peri-implant diseases and conditions organized at the 2017 World Workshop on Periodontics [14]..

According to this classification;

- Healthy periodontium: Individuals with no clinical attachment loss and bleeding on probing less than 10%.
- Gingivitis: Individuals with no clinical attachment loss and bleeding on probing greater than 10%.
- Stage I periodontitis: Maximum CAL 1–2 mm, radiographic bone loss 15–33% of the coronal third.
- Stage II periodontitis: Maximum CAL 3–4 mm, radiographic bone loss 15–33% of the coronal third.

### Statistical analysis

All statistical analyses were conducted using SPSS v26.0 (IBM Corp., Armonk, NY, USA).The normality of the data was assessed using the Kolmogorov-Smirnov test. Continuous variables showing a normal distribution were presented as mean ± standard deviation, and an independent samples t-test was used for intergroup comparisons. Variables that did not show a normal distribution were expressed as median (interquartile range) and analyzed using the Mann-Whitney U test. For categorical variables, n (%) values were provided and the Chi-square or Fisher’s exact test was used.the level of statistical significance was accepted as *p* < 0.05. Bonferroni correction was applied for multiple comparisons involving periodontal parameters.

**Table 1:** Demographic and clinical characteristics of patients with polymyalgia rheumatica and control groups

	PMR (*n* = 48)	Control (*n* = 52)	*p* value
Sex ( male/female)	(7) 14.6% / (41) 85.4%	(10)19.2% /(42)80.8%	0.541
Age (years, mean±SD)	57.8±8.68	53.8±10.49	0.41
ESR, mm/h (mean±SD)	62.7±10.06	8.3 ± 3.1	<0.001
CRP, mg/L (mean±SD)	74.7±9.23	2.1 ± 0.9	<0.001
PMR-AS	23 ± 4	-	

*PMR*, polymyalgia rheumatica; *ESR*, erythrocyte sedimentation rate; *CRP*, C reactive protein; *PMR-AS*, polymyalgia rheumatica activity score

## Results

The study group consisted of 48 patients with PMR (Male/Female: 7/41), while the control group comprised 52 patients (Male/Female: 10/42). There was no difference in terms of gender between the two groups (*p* = 0.541). In the PMR group, the average age was found to be 57 ± 8, while in the control group it was 53 ± 10, with no significant difference between them (*P* = 0.41). As expected, inflammatory markers such as CRP and erythrocyte sedimentation rate were significantly higher in the PMR group compared to controls (both *P* < 0.001). The demographic characteristics of the patient and control groups are shown in Table 1. Among the 48 PMR patients, 11 had Stage I periodontitis, 3 had Stage II periodontitis, 30 had gingivitis, and 4 were healthy. In the 52 healthy Control group; 9 had Stage I periodontitis, 3 had Stage II periodontitis, 34 had gingivitis, and 6 were healthy. Although no statistically significant difference was found between the PPD, CAL, and PI values, the PPD and CAL values were higher in the PMR group. When comparing BoP values, the PMR group was found to have 58.3 ± 34.7%, while the control group had 37.8 ± 25.3%, and the difference was statistically significant (*p* = 0.001). When comparing GI, it was found to be significantly higher in PMR patients compared to the healthy control group (*p* = 0.007).The periodontal disease indices of the patient and control groups are shown in Table 2.

**Table 2:** The periodontal disease indices of the patient and control groups

	Polymyalgia rheumatica (*n* = 48)	Healthy control (*n* = 52)	*P* value
Periodontitis frequency, %(n)	(14) 29.1%	(12) 23%	0.724
CAL	2.78 ±0.99	2.48±0.78	0.98
PPD	2.60 ±0.88	2.31±0.62	0.58
PI	1.46±0.56	1.52±1.59	0.607
GI	1.56±0.50	1.27±0.54	0.007
BoP	58.31±34.71	37.82±25.30	0.001

*CAL*, clinical attachment level; *PPD*, probing pocket depth; *PI*, plaque index; *GI*, gingival index; *BoP*, bleeding on probing

## Discussion

This study is the first in the literature to examine the relationship between polymyalgia rheumatica (PMR) and periodontal diseases. Our findings indicate that periodontal inflammation is significantly increased in patients with PMR. We found that the gingival index (GI) (*p* = 0.007) and bleeding on probing (BoP) (*p* = 0.001) values were statistically significantly higher in the PMR group compared to healthy controls. These data suggest that the systemic inflammatory nature of PMR may affect periodontal tissues.

PMR is a systemic inflammatory disease characterized by an increase in pro-inflammatory cytokines. As shown in the foundational study by Weyand et al. [15], proinflammatory cytokines such as IL-6, IL-1β, and TNF-α play a significant role in the pathogenesis of PMR. In the current literature, Weyand and Goronzy [2] have contributed to the understanding of immune mechanisms in PMR by emphasizing the role of these cytokines in the pathogenesis of vasculitis. Similarly, Preshaw and Taylor [5] have shown the role of the same cytokines in the pathogenesis of periodontal disease.

Our findings support the possible influence of systemic inflammatory load on the periodontium. The role of inflammatory mediators in the development of periodontal diseases may also play a role in the pathogenesis of PMR, which could explain the biological relationship between these two conditions.

Interestingly, while there was no significant difference in the plaque index (PI) values, which is the primary etiological factor for periodontal tissues, between the groups (*p* = 0.6), a significant difference was found in the gingival index (GI) values, which indicate soft tissue inflammation (*p* = 0.007). This situation suggests that PMR could be a systemic predisposing factor in addition to local factors in periodontal inflammation. Similarly, the significantly higher bleeding on probing rate in the PMR group (%58.3 ± 34.7 vs. %37.8 ± 25.3, *p* = 0.001) suggests a possible effect of PMR on periodontal vascular structures. The observation that plaque index values were comparable between groups, while inflammatory parameters such as GI and BoP were significantly elevated in PMR patients, suggests that periodontal inflammation in this population may not be solely driven by local factors. Instead, systemic inflammation associated with PMR may play a contributory role in amplifying gingival inflammatory responses.

In our study, PPD (Probing Pocket Depth) values in the PMR group were not found to be statistically significantly different compared to healthy controls (*p* = 0.58), although numerically higher values were observed in the PMR group (2.60 ± 0.88 vs. 2.31 ± 0.62). Additionally, while there was no statistically significant difference in CAL (Clinical Attachment Level) values, numerically higher values were noted in the PMR group (2.78 ± 0.99 vs. 2.48 ± 0.78, *p* = 0.98). These findings suggest a potential relationship between PMR and tissue destruction in the periodontium.

The activation of matrix metalloproteinases (MMPs), which play a role in the progression of periodontal disease, is under the control of inflammatory cytokines such as TNF-α and IL-1β [5]. In PMR, increased systemic cytokines are likely to contribute to periodontal tissue destruction by inducing MMP activation. Sezer and colleagues [16] have shown that chronic periodontitis increases oxidative stress parameters in patients with rheumatoid arthritis (RA).

This situation suggests that oxidative stress may also contribute to periodontal tissue destruction in RA and similarly in PMR patients.

The lack of a statistically significant difference in the prevalence of periodontitis between the PMR group and the control group (%29.1 vs. %23, *p* = 0.724) can be interpreted as either being in the early stage of the disease or the sample size being insufficient to detect this difference. The findings of our study are consistent with the periodontal involvement observed in other systemic inflammatory diseases. Recent studies have further highlighted the bidirectional relationship between periodontal inflammation and systemic inflammatory conditions, emphasizing the role of shared inflammatory pathways and immune responses [17]. In the literature, it has been reported that the prevalence of periodontal disease increases in systemic inflammatory diseases such as rheumatoid arthritis [18], psoriatic arthritis [6], Behçet’s disease, and familial Mediterranean fever [7]. These studies support the negative impact of systemic inflammation on periodontal tissues. Ceccarelli et al. [18] have thoroughly examined the common inflammatory mediators between RA and periodontitis and have revealed the potential pathophysiological connections between these two diseases. Similarly, Üstün and colleagues [6] reported the prevalence of periodontal disease in patients with psoriatic arthritis as 29.1%, which is similar to the prevalence we found in PMR patients in our study. Erciyas and colleagues [19] have shown that periodontal treatment reduces systemic inflammation markers and disease activity in RA patients. This study is important in highlighting the potential positive effects of periodontal treatment on systemic inflammatory diseases. The presence of a similar effect in PMR patients is worth investigating.

From a clinical perspective, monitoring periodontal status in PMR patients could provide complementary insights into systemic disease activity. This highlights the importance of interdisciplinary management involving both rheumatologists and periodontists. However, it should be emphasized that the cross-sectional nature of our study prevents conclusions about causality. Longitudinal studies assessing the temporal relationship between systemic inflammation and periodontal outcomes in PMR are warranted.

Our study has some limitations. First, due to the cross-sectional design, a causal relationship cannot be established. To understand the temporal dimension of the relationship between PMR and periodontal disease, prospective, longitudinal studies are needed. Secondly, the sample size is limited, and studies with larger cohorts need to be conducted. Thirdly, the failure to measure cytokine levels in gingival crevicular fluid and serum prevents a complete elucidation of the pathophysiological mechanisms. Another limitation is the lack of detailed data on oral hygiene practices such as toothbrushing frequency, dental visits, or use of adjunctive oral care products. Another limitation of this study is the absence of a priori sample size calculation, which may have limited the statistical power to detect differences between groups.

In conclusion, this study shows that periodontal inflammation is significantly increased in patients with PMR. Especially the significantly higher levels of periodontal parameters related to soft tissue inflammation, such as GI and BoP, in PMR patients support the impact of systemic inflammation on periodontal tissues. The increase in PPD values suggests that more advanced periodontal tissue destruction may develop over time. As a result, the increased risk of periodontal disease in PMR patients should be kept in mind, and these patients should be closely monitored for periodontal evaluation and treatment.

## Data Availability

The raw patient data used in this study cannot be made publicly available due to ethics committee requirements and patient confidentiality. However, an anonymized dataset with all identifying information removed can be provided by the corresponding author upon reasonable request during the peer-review process or after publication.
